# Association between psychosis and substance use in Kenya. Findings from the NeuroGAP-Psychosis study

**DOI:** 10.3389/fpsyt.2024.1301976

**Published:** 2024-02-29

**Authors:** Monica Nguata, James Orwa, Gabriel Kigen, Edith Kamaru, Wilfred Emonyi, Symon Kariuki, Charles Newton, Linnet Ongeri, Rehema Mwende, Stella Gichuru, Lukoye Atwoli

**Affiliations:** ^1^ Department of Post Traumatic Stress Disorder (PTSD) Tnx, Academic Model Providing Access to Healthcare (AMPATH), Eldoret, Kenya; ^2^ Department of Population Health, Medical College of East Africa Aga Khan University, Nairobi, Kenya; ^3^ Department of Pharmacology and Toxicology, Moi University School of Medicine, Eldoret, Kenya; ^4^ Department of Mental Health, Moi Teaching and Referral Hospital, Eldoret, Kenya; ^5^ Department of Immunology, Moi University School of Medicine, Eldoret, Kenya; ^6^ Neuroscience Unit, Kenya Medical Research Institute (KEMRI) Wellcome Trust Research Programme, Kilifi, Kenya; ^7^ Department of Public Health, Pwani University, Kilifi, Kenya; ^8^ Department of Psychiatry, University of Oxford, Oxford, United Kingdom; ^9^ Department of Medicine, Medical College East Africa, the Aga Khan University, Nairobi, Kenya; ^10^ Department of Mental Health and Behavioural Sciences, Moi University School of Medicine, Eldoret, Kenya; ^11^ Brain and Mind Institute, the Aga Khan University, Nairobi, Kenya

**Keywords:** cases, controls, psychosis, schizophrenia, bipolar mood disorders, substance use disorders

## Abstract

**Background:**

Substance use is prevalent among people with mental health issues, and patients with psychosis are more likely to use and misuse substances than the general population. Despite extensive research on substance abuse among the general public in Kenya, there is a scarcity of data comparing substance use among people with and without psychosis. This study investigates the association between psychosis and various substances in Kenya.

**Methods:**

This study utilized data from the Neuro-GAP Psychosis Case-Control Study between April 2018 and December 2022. The KEMRI-Wellcome Trust Research Programme recruited participants from various sites in Kenya, including Kilifi County, Malindi Sub-County, Port Reitz and Coast General Provincial Hospitals, and Moi Teaching and Referral Hospital, as well as affiliated sites in Webuye, Kapenguria, Kitale, Kapsabet, and Iten Kakamega. The collected data included sociodemographic information, substance use, and clinical diagnosis. We used the summary measures of frequency (percentages) and median (interquartile range) to describe the categorical and continuous data, respectively. We examined the association between categorical variables related to psychosis using the chi-square test. Logistic regression models were used to assess the factors associated with the odds of substance use, considering all relevant sociodemographic variables.

**Results:**

We assessed a total of 4,415 cases and 3,940 controls. Except for alcohol consumption (p-value=0.41), all forms of substance use showed statistically significant differences between the case and control groups. Cases had 16% higher odds of using any substance than controls (aOR: 1.16, 95%CI: 1.05-1.28, p=0.005). Moreover, males were 3.95 times more likely to use any substance than females (aOR:3.95; 95%CI: 3.43-4.56). All the categories of living arrangements were protective against substance use.

**Conclusion:**

The findings of this study suggest that psychotic illnesses are associated with an increased likelihood of using various substances. These findings are consistent with those of previous studies; however, it is crucial to investigate further the potential for reverse causality between psychosis and substance abuse using genetically informed methods.

## 1 Introduction

Substance abuse has emerged as a pressing global public health issue. One of the primary concerns is the rising incidence of substance abuse comorbid with psychotic illnesses such as Schizophrenia Spectrum Disorder and Bipolar Mood Disorder. According to the World Health Organization, psychotic illness ranks as the 22nd leading cause of disability worldwide (1). Substance abuse, as defined by the World Health Organization, refers to the dangerous or detrimental use of psychoactive substances, including alcohol and illicit drugs (2). Psychosis is characterized by a constellation of symptoms that lead to a disrupted connection with reality. Among these symptoms are hallucinations and delusions, which are frequently observed in Patients diagnosed with a schizophrenia spectrum disorder. Conversely, patients diagnosed with bipolar mood disorder may experience depressive and manic or mixed episodes as psychotic symptoms. It is generally recognized that substance abuse is more common among those with psychotic illnesses (3).

The interrelationship between substance abuse and psychosis is intricate and multifaceted, as both conditions involve disturbances in brain function and can have a significant impact on an individual’s mental health. To understand this relationship, it is crucial to consider various factors, such as the nature of the substances involved, individual susceptibilities, and the underlying neurobiological mechanisms.

Previous research suggests that co-occurring substance use disorders are prevalent among individuals with psychotic disorders, with a reported incidence of up to 50% (4). This figure is notably higher than the prevalence of these disorders in the general population. In the United States, a study among patients with schizophrenia indicated that these individuals were approximately 4.6 times more likely to engage in substance use and misuse than the general population (5). Furthermore, a survey revealed that the prevalence of lifetime alcohol and substance use disorders in the general population was approximately 17%. In contrast, the rates were significantly higher in patients with schizophrenia (47%), bipolar mood disorder (56%), and anxiety disorder (30%) (6). Another study found a strong correlation between schizophrenia and bipolar mood disorder diagnosis and substance abuse. The study population also demonstrated a higher prevalence of substance use than similar research conducted in other settings. However, male sex was identified as a significant predictor of substance addiction for all substances (7).

The motivation for substance use among patients with psychosis is not yet clearly understood. However, the concept known as maladaptive coping has been associated with the use of several substances in the patient population (8). Patients diagnosed with psychosis are more likely to resort to maladaptive coping mechanisms than the general population. These coping strategies include passive, comfortable, and avoidant coping strategies (9). In the general population, maladaptive coping is more prevalent in smokers than in nonsmokers (10). According to a study, patients with schizophrenia often use drugs to relieve negative symptoms such as dysphasia and depression (7). Furthermore, these substances enhanced social interactions and alleviated anxiety and dysphoria. The precise reasons for substance abuse in patients with bipolar disorder remain unclear. However, A study revealed that dampened response to a low dose of psychostimulant drugs among patients with a history of elevated mood (Hypomania) may increase the risk of subsequent drug use or misuse (11). Additionally, it has been suggested that one of the diagnostic criteria for mania, a characteristic feature of bipolar disorder, is excessive engagement in pleasurable activities that may result in harmful consequences. Consequently, patients with bipolar disorder are known to exhibit heightened substance use during manic episodes (12).

A survey conducted nationwide in Kenya in 2017 demonstrated that a substantial proportion of the population aged 15–65 years suffered from substance use disorders, with more than 10% of the population affected. Another survey revealed that 20% of school-going children used at least one substance at some point in their lives (13). Ndetei reported a high incidence of alcohol abuse and dependence on opiates, sedatives, khat use, mood disorders, and other psychotic disorders (14). Despite extensive research on substance abuse among the general public in Kenya, there is a lack of data comparing substance use among individuals with and without psychosis. Establishing disparities in substance use between individuals with psychosis (cases) and those without (controls) is crucial to guide future initiatives and effectively address substance use and psychosis in Kenya.

## 2 Participants and methods

### 2.1 Study participants

These analyses used the Kenya data from the Neuropsychiatric Genetics of African Populations-Psychosis (NeuroGAP-Psychosis) study collected from April 2018 to December 2022. NeuroGAP-psychosis was a multi-country case-control and genome-wide association study (GWAS) to deepen understanding of genetic and environmental risk factors for psychotic disorders in Africa. A full explanation of the methodology of the NeuroGAP-Psychosis study is detailed elsewhere (15). Over 4 years, the study team recruited a total of 4,415 schizophrenia and Bipolar Mood Disorder (cases) and 3,940 controls across two Kenyan sites. Participants enrolled in NeuroGAP-Psychosis were recruited from the Moi Teaching and Referral Hospital and affiliated sites in Webuye, Kapenguria, Kitale, Kapsabet, Iten Kakamega; and the KEMRI-Wellcome Trust Research Programme recruiting sites (Kilifi County, Malindi sub-County, Port Reitz and Coast General Provincial Hospitals). Our analysis was limited to data from Kenya between April 2018 and the end of 2022, as documented in other sources (14).

### 2.2 Cases and controls

**Figure 1:**
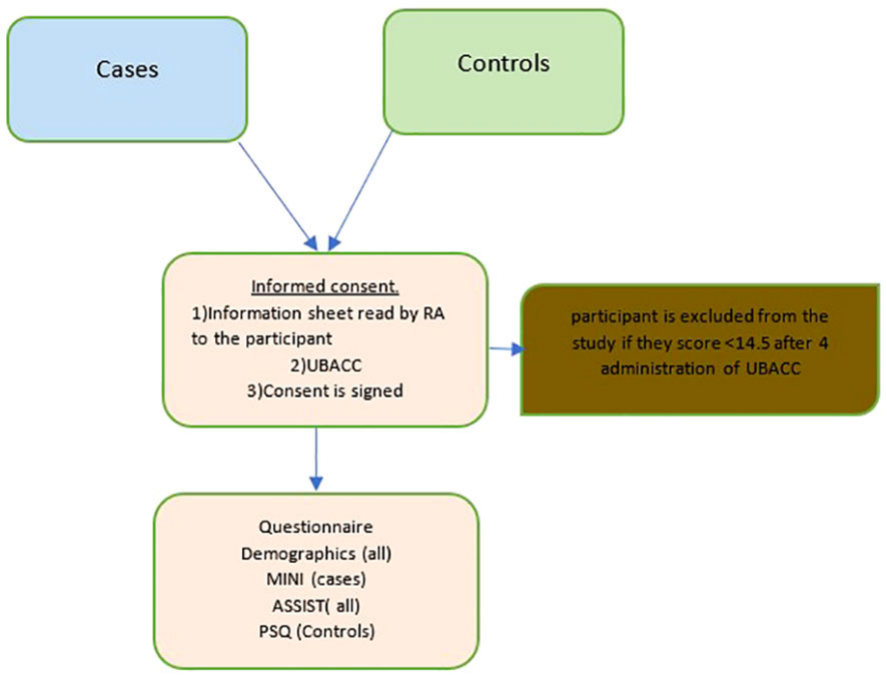
The study process for cases and controls. ASSIST, Alcohol, Smoking and Subtance Involvement Screening Test, MINI, Mini International Neuropsychiatric Interview, Standard 7.0.2 for Diagnostic and Statistical of Mental Disorder-5, PSQ, Psychosis Screening Questionnaire; RA, research assistant; UBACC, University of California, San Diego Brief Assesment of Capacity to Consent.

The control group participants comprised People seeking outpatient general medical care, accompanying friends or family members to clinic visits, hospital/clinic staff, or those attending for other reasons, such as collecting a prescription refill. The cases included People with a clinical diagnosis of psychosis confirmed by either clinician referral or medical record review. The grouping of Schizophrenia and Bipolar I mood disorders as psychotic disorders was based on the literature that showed a significant genetic correlation between the two conditions, suggesting that some genetic variants may increase the risk of both phenotypes (15). To confirm the diagnosis, the Mini International Neuropsychiatric Interview (MINI) was administered to the cases. All participants, including those in the control and case groups, were required to be at least 18 years old and provide written informed consent or a fingerprint in cases of illiteracy. The cases were matched to controls based on age and gender using the University of California, San Diego Brief Assessment of Capacity to Consent (UBACC) tool, which was utilized in South Africa with comparable demographics and served as an interactive learning tool. Specific details of the exclusion criteria have been provided elsewhere (15). (See **Figure 1**)

### 2.3 Assessment instruments

#### 2.3.1 Substance use

To determine the frequency of substance use, The WHO ASSIST (Alcohol, Smoking, and Substance Involvement Screening Test) (16) was administered to both cases and controls. This tool was developed by an international group of researchers and clinicians to assist with the early identification of substance use-related health risks and substance use disorders in primary health care, general medical care, and other settings. The study utilized only the first part of the tool as it sought to just establish the frequency of use for different substances. In this study, “substance use” referred to the non-medical use of any substance falling into categories of the ASSIST over the past 3 months before the study. These substances were: alcoholic beverages, tobacco products, cannabis, cocaine, amphetamine-type stimulants, inhalants, sedatives, hallucinogens, opioids, and others. These definitions have been previously used in other studies (17, 18). The frequency of use was grouped into two categories. ‘Daily/Almost Daily or Weekly’ was categorized as ‘Frequent use’ while ‘once or twice and Monthly’ was grouped as ‘None-Frequent use.’

#### 2.3.2 Psychosis assessment tools (MINI)

The Mini-International Neuropsychiatric Interview (MINI) is a brief, structured diagnostic tool for DSM-IV and ICD-10 psychiatric illnesses. It was developed for a quick reliable structured psychiatric interview for multi-center clinical trials and epidemiology studies (19). It can be used as the first step in outcome tracking in non-clinical research settings. This tool was administered to cases only to supplement the clinical diagnosis of psychosis. The details of its administration are found elsewhere (15)

#### 2.3.3 The psychosis screening questionnaire

This was administered to controls to match MINI in the cases. The Psychosis Screening Questionnaire (PSQ) is a quick tool for diagnosing psychotic illnesses. The scale was created to test for the presence of recent psychotic symptoms. It asks for hypomania, thought insertion, paranoia, thought insertion, and hallucinations in five probing questions. Each part includes a follow-up question if the respondent’s response is positive (20) The positive controls were retained as controls despite scoring for positive symptoms. This is because we used a screening tool rather than a clinical diagnosis. They did not meet the inclusion criteria for cases because the criterion for inclusion was a confirmed clinical diagnosis of psychosis from patient records before enrolment. However, these patients were subsequently referred for clinical diagnosis.

#### 2.3.4 Socio-demographic questionnaire

The study participants provided sociodemographic information, including age, gender, marital status, place of birth, current residence, living arrangements, educational attainment (whether they had completed college), and patient type.

### 2.4 Statistical analysis

All analyses were conducted utilizing RStudio version 4.1.3 (2022–03–10). Demographic and other study variables were summarized using frequencies, percentages, medians, and corresponding interquartile ranges (IQR). Pearson’s Chi-squared or Fisher’s exact test was used to compare the frequency of categorical variables, including abused substances, between cases and controls. Continuous variables were compared using the Wilcoxon rank-sum test. Univariable and multivariable logistic regression analyses were performed to assess the risk factors associated with any substance use and to quantify the association using the odds ratio and the corresponding 95% confidence interval (CI). Our modeling approach was most effectively described by the use of multivariable or multiple logistic regression, as our structure comprised a single outcome variable and several independent or predictor variables (21). Patient type and all sociodemographic factors were included in the multivariable analysis *a priori*, as they are considered relevant in explaining the risk of substance use. Statistical significance was set at P < 0.05.

## 3 Results

As shown in **Table 1**, the total number of participants, 3940 controls, and 4415 Cases met the inclusion criteria. There was no significant difference in the age and sex of the respondents between the two groups. The proportion of females was higher among the controls than in cases (47.9% vs. 45.9%; p-value=0.060). The Median age in years for controls was 34 (26.0-44.0) while the one for cases was 34.0 (26.0 – 43.0). Among the controls, 2,089 (53.0) were married compared to 1,592 (36.1) (p-value<0.001). The majority (n=1298; 33.0%) of the controls had finished college, while among the cases majority had finished the primary level of education (n=1112, 25.2%). There was a significant difference in living arrangements between the cases and the controls. The majority (1,899 (48.2) while the majority of the cases lived with parental family (1,900 (43.0) (**Table 1**).

**Table 1:** Socio-demographic characteristics of participants by cases and control.

Variable	Patient Type		p-value^2^
	control, N = 3,940^1^	case, N = 4,415^1^	
**Age in years, Median (IQR)**	34.0 (26.0 – 44.0)	34.0 (26.0 – 43.0)	0.44
**age group, n (%)**			0.76
18-35	2,154 (54.7)	2,439 (55.2)	
36-55	1,497 (38.0)	1,644 (37.2)	
56-83	289 (7.3)	332 (7.5)	
**Sex at birth, n (%)**			0.060
Females	1,889 (47.9)	2,026 (45.9)	
Males	2,051 (52.1)	2,389 (54.1)	
**Marital Status, n (%)**			<0.001
Married/in-union	2,089 (53.0)	1,592 (36.1)	
Widowed/Divorced or annulled/Separated/Other	479 (12.2)	906 (20.5)	
Never been married	1,372 (34.8)	1,917 (43.4)	
**Level of education, n (%)**			<0.001
No education	44 (1.1)	54 (1.2)	
Some primary school	285 (7.2)	666 (15.1)	
Finished primary school	574 (14.6)	1,112 (25.2)	
Some secondary school	297 (7.5)	592 (13.4)	
Finished secondary school	890 (22.6)	987 (22.4)	
Some college	551 (14.0)	344 (7.8)	
Finished college	1,298 (33.0)	659 (14.9)	
**Living arrangement, n (%)**			<0.001
Lives alone	739 (18.8)	423 (9.6)	
Lives with parental family	727 (18.5)	1,900 (43.0)	
Lives with spouse or partner	1,899 (48.2)	1,393 (31.6)	
Lives with other relatives	574 (14.6)	698 (15.8)	

^1^Median (IQR) or Frequency (%).

^2^Wilcoxon rank sum test; Pearson’s Chi-squared test.

**Table 2** presents alcohol and substance use characteristics of cases and controls. Except for alcohol consumption (p-value=0.41), all substance use between the case and controls differed significantly. Products like tobacco, Khat, and marijuana were more prevalent in use cases than in controls (p-value = 0.001). Despite the low numbers, considerably more cases than controls took sedatives and/or opioids (p-value=0.001). More controls than cases used sedatives or sleeping pills (p-value=0.001). Between the cases and controls, there was no significant variance in the usage of any substance (p-value=0.011). (**Table 2**)

**Table 2:** Alcohol and substance use characteristics of cases and controls.

Variable	Patient Type		p-value^2^
	control, N = 3,940^1^	case, N = 4,415^1^	
**Tobacco Products (cigarettes, chewing tobacco, cigars, etc.), n (%)**	682 (17.3)	1,387 (31.4)	<0.001
**Tobacco Products: How often have you used these substances in the past 3 months?, n (%)**			<0.001
None	413 (60.6)	712 (51.3)	
None frequent	86 (12.6)	235 (16.9)	
Frequent	183 (26.8)	440 (31.7)	
**Alcoholic beverages (beer, wine spirits, homebrew, etc), n (%)**	1,811 (46.0)	1,990 (45.1)	0.41
**Alcohol Products: How often have you used these substances in the past 3 months?, n (%)**			<0.001
None	947 (52.3)	1,358 (68.2)	
None frequent	636 (35.1)	376 (18.9)	
Frequent	228 (12.6)	256 (12.9)	
**Khat products, n (%)**	485 (12.3)	976 (22.1)	<0.001
**Khat Products: How often have you used these substances in the past 3 months?, n (%)**			0.068
None	261 (53.8)	578 (59.2)	
None frequent	124 (25.6)	240 (24.6)	
Frequent	100 (20.6)	158 (16.2)	
**Cannabis (marijuana, pot, grass, hash, etc.), n (%)**	454 (11.5)	991 (22.4)	<0.001
**Cannabis Products: How often have you used these substances in the past 3 months?, n (%)**			0.094
None	291 (64.1)	692 (69.8)	
None frequent	91 (20.0)	169 (17.1)	
Frequent	72 (15.9)	130 (13.1)	
**Other over-the-counter meds (antihistamines, cough syrups), n (%)**	88 (2.2)	46 (1.0)	<0.001
**Other Over-the-Counter Meds: How often have you used these substances in the past 3 months?, n (%)**			0.014
None	36 (40.9)	31 (67.4)	
None frequent	41 (46.6)	12 (26.1)	
Frequent	11 (12.5)	3 (6.5)	
**Sedatives or Sleeping Pills (Valium, mandrax/methaqualone, sere-pax, Rohypnol, etc.), n (%)**	44 (1.1)	138 (3.1)	<0.001
**Opioids (heroin, morphine, oxycontin, methadone, codeine, etc.), n (%)**	28 (0.7)	144 (3.3)	<0.001
**Opioids Products: How often have you used these substances in the past 3 months?, n (%)**			<0.001
None	10 (35.7)	128 (88.9)	
None frequent	3 (10.7)	4 (2.8)	
Frequent	15 (53.6)	12 (8.3)	
**Any substance use, n (%)**	1,986 (50.4)	2,348 (53.2)	0.011

^1^Median (IQR) or Frequency (%).

^2^Pearson’s Chi-squared test; Fisher’s exact test.

**Table 3** shows the univariable and multivariable logistic regression analysis. The bivariate analysis showed that being in the 56-83 age group, males, widowed/divorced or annulled/separated/other type of marital status, living arrangements, and being a case were statistically significant factors for any substance use. However, in the multivariable analysis, any substance use was significantly associated with being a male, having never been married, level of education (finished primary school, some secondary school, and some secondary school), living arrangements, and patient type. Males had five times increased odds of using any substance compared to females (aOR: 5.07, 95%CI:4.06-5.60, p < 0.001). Similarly, cases had 16% higher odds of using any substance as compared to controls (aOR: 1.16, 95%CI: 1.05-1.28, p=0.005). Participants who had never been married were 45% less likely to use substances compared to those who are married/in-union (aOR: 0.55, 95%CI: 0.43-0.69); those who had finished primary education, some secondary, and those who had finished secondary school had a reduced odds of using any substance than those who with no level of education. There were also reduced odds of substance use among participants who lived with the parental family, with spouse or partner, and those who lived with other relatives compared to those who lived alone.

**Table 3:** shows the univariable and multivariable logistic regression analysis.

Variables	Categories	Any Substance use		Univariable		Multivariable	
		No	Yes	OR (95%CI)	p-value	aOR (95%CI)	p-value
		n (%)	n (%)				
Age-group	18-35 years	2216 (48.2)	2377 (51.8)		Reference		Reference
	36-55 years	1544 (49.2)	1597 (50.8)	0.96 (0.88-1.06)	0.432	0.96 (0.86-1.07)	0.470
	56-83 years	261 (42.0)	360 (58.0)	1.29 (1.09-1.52)	0.004	1.19 (0.98-1.45)	0.087
Sex	Females	2628 (67.1)	1287 (32.9)		Reference		Reference
	Males	1393 (31.4)	3047 (68.6)	4.47 (4.07-4.90)	<0.001	5.07 (4.60-5.60)	<0.001
Marital status	Married/in-union	1825 (49.6)	1856 (50.4)		Reference		Reference
	Widowed/Divorced or annulled/Separated/Other	634 (45.8)	751 (54.2)	1.16 (1.03-1.32)	0.016	1.01 (0.79-1.30)	0.915
	Never been married	1562 (47.5)	1727 (52.5)	1.09 (0.99-1.19)	0.082	0.55 (0.43-0.69)	<0.001
Education	No education	46 (46.9)	52 (53.1)		Reference		Reference
	Some primary school	428 (45.0)	523 (55.0)	1.08 (0.71-1.64)	0.714	0.81 (0.52-1.25)	0.338
	Finished primary school	819 (48.6)	867 (51.4)	0.94 (0.62-1.41)	0.753	0.59 (0.38-0.92)	0.019
	Some secondary school	453 (51.0)	436 (49.0)	0.85 (0.56-1.29)	0.451	0.55 (0.35-0.86)	0.009
	Finished secondary school	934 (49.8)	943 (50.2)	0.89 (0.59-1.34)	0.586	0.54 (0.35-0.84)	0.006
	Some college	428 (47.8)	467 (52.2)	0.97 (0.63-1.47)	0.868	0.70 (0.45-1.11)	0.127
	Finished college	913 (46.7)	1044 (53.3)	1.01 (0.67-1.52)	0.956	0.75 (0.48-1.16)	0.189
Living arrangements	Lives alone	396 (34.1)	766 (65.9)		Reference		Reference
	Lives with parental family	1286 (49.0)	1341 (51.0)	0.54 (0.47-0.62)	<0.001	0.59 (0.50-0.69)	<0.001
	Lives with spouse or partner	1677 (50.9)	1615 (49.1)	0.50 (0.43-0.57)	<0.001	0.36 (0.28-0.47)	<0.001
	Lives with other relatives	662 (52.0)	610 (48.0)	0.48 (0.40-0.56)	<0.001	0.60 (0.50-0.72)	<0.001
Patient type	Control	1954 (49.6)	1986 (50.4)		Reference		Reference
	Case	2067 (46.8)	2348 (53.2)	1.12 (1.03-1.22)	0.011	1.16 (1.05-1.28)	0.005

aOR, Adjusted odds ratio.

## 4 Discussion

### 4.1 Main findings

The current investigation evaluated the association between psychosis and the use of various substances in Kenya. The results revealed that psychosis was linked to increased odds of substance use. This is consistent with previous studies that showed higher rates of substance use among patients with psychosis than among the general population (3, 6, 22). This correlation may be attributed to dysregulation of reward circuits or neurochemical abnormalities in psychosis, which could increase the likelihood of developing substance use disorders (23, 24).

Although the frequency of tobacco use was higher in the case group than in the control group, the difference was not statistically significant. This could be attributed to the fact that maladaptive behavior in smokers is common, regardless of whether they have psychosis as suggested by previous studies (25, 26). Therefore, it is essential to pay close attention to the general public as it is to patients with psychosis.

Our research contradicted the results of previous studies which found that Patients with psychosis consume alcohol at almost twice the rate of those without psychosis (6, 27). The underlying reasons for this disparity are complex and multifaceted. However, several studies have shed light on the link between alcohol consumption and psychosis. Two studies indicated that patients may turn to alcohol as a means of counteracting these adverse effects or regaining pleasurable experiences (6, 28). A study to establish these theories is recommended.

The frequency of Khat use was found to be twice as high in cases than in controls. This may be attributed to the fact that Khat is legal in Kenya and deeply ingrained in cultural and social practices (29, 30). Additionally, Khat may be used more frequently as a substitute for other stimulants, such as amphetamines and cocaine. Although our study demonstrated higher cannabis use in cases than in controls, previous studies suggest that there is a dose-dependent risk of developing psychotic illness with cannabis use. Cannabis users are known to experience psychotic disease onset at an earlier age than non-users (31). It is however difficult to determine whether this is because of reverse causality. Further studies are needed to distinguish between these two.

Although sedatives and opioids were used less frequently, they were the second most commonly used substances among cases. This is consistent with the results of other studies (6). The prevalence of substance use among study participants was relatively low, and additional research is recommended to substantiate these findings. Moreover, no statistically significant disparity was observed in the substance use patterns between the case and control groups.

Our study revealed that controls used over-the-counter (OTC) medications more frequently than cases; however, there is a lack of data to compare our findings with those of other studies. Additionally, our results may be subject to bias because a portion of the control population was sourced from the hospital environment. These People may have been accompanied by a family member at the hospital or had come in for a check-up and were self-medicating with OTC drugs. Further research examining the frequency and types of OTC drugs used would provide valuable insights.

Although substance use was more prevalent among cases than controls, the most significant predictors for both groups were male sex and living arrangement, which is consistent with previous studies (32). Access accounts for much of the gender difference in the prevalence of substance use, and men are more likely to access substances than women. Additionally, women are also more susceptible to the negative health and psychosocial consequences of substance use (33–35). The extent of sex differences in various substances remains uncertain, but some studies have indicated potentially significant variations. A deeper understanding of sex and sex differences is needed to develop tailored prevention and intervention programs for each population.

In our observations, we found a correlation between people who had never been married and those who had not. This correlation can be explained by the social support, stability, and accountability offered by marital relationships. This can contribute to healthier behaviors and decreased substance use. The absence of social support can increase feelings of loneliness, making Patients more susceptible to using substances as compensation. Furthermore, loneliness can exacerbate difficulties experienced by Patients living with psychosis. Substance abuse may offer a reprieve or a coping mechanism for managing overwhelming emotions and thoughts.

In both cases and controls, no association was observed between level of education and substance use. We believe that education level by itself may not adequately encompass the complete spectrum of factors that contribute to substance use. People with the same educational background may have disparate experiences, motivations, and backgrounds. Personality traits, mental health conditions, and genetic predispositions may be more significant than educational level in influencing substance use.

Our finding on the living arrangement was nearly identical in association with substance use for both cases and controls. Social support is known to provide an in-built support system. It reduces feelings of isolation and increases responsibility and accountability, thus building on healthy coping strategies in cases of distress. These cut across all humanity regardless of their mental health state. This finding suggests that living arrangements could be one of the most powerful preventive tools for substance abuse, and more research could bring insight into this topic.

### 4.2 Strengths and limitations

The case and control sample sizes are relatively large and therefore substantial in representation of the population in Kenya. This study however had several limitations. The current study’s cross-sectional methodology precludes the evaluation of the short- and long-term effects of psychosis on substance use. Moreover, the sample of relatively high-functioning patients is included in the cohort; hence, maladaptive substance use may have been underestimated. This cross-sectional analysis and observational design make it impossible to rule out reverse causality and residual confounding completely. Finally, although some controls had positive symptom scores, they remained in the control group. Thus, it is impossible to factor in the potential for a positive symptom bias about substance use.

## 5 Conclusion

Our study revealed that people with psychotic disorders were more likely to use substances than the general population, with Alcohol and Khat consumption being the most common. Male sex and living arrangements were also linked to substance use. While these findings align with those of previous studies, reverse causality between psychosis and substance abuse should be explored further using genetically informed methods. Furthermore, it is imperative to create tailored treatment plans that prioritize addressing both psychotic and co-occurring substance use disorders in patients with these conditions.

## Data availability statement

The raw data supporting the conclusions of this article will be made available by the authors, without undue reservation.

## Ethics statement

The studies involving humans were approved by The Kenya National Council of Science and Technology (#NACOSTI/P/17/56302/19576), the Moi University College of Health Sciences/Moi Teaching and Referral Hospital Institutional Research and Ethics Committee (#IREC/2016/145, approval number: IREC 1727), the KEMRI Center Scientific Committee (CSC# KEMRI/CGMRC/CSC/070/2016), and the KEMRI Scientific and Ethics Review Unit (SERU#KEMRI/SERU/CGMR-C/070. The studies were conducted in accordance with the local legislation and institutional requirements. The participants provided their written informed consent to participate in this study.

## Author contributions

MN: Writing – original draft. JO: Formal analysis, Writing – review & editing. GK: Writing – review & editing. EK: Writing – review & editing. WE: Writing – review & editing. SK: Writing – review & editing. CN: Conceptualization, Writing – review & editing. LO: Writing – review & editing. RM: Project administration, Writing – review & editing. SG: Project administration, Writing – review & editing. LA: Conceptualization, Funding acquisition, Supervision, Writing – review & editing. JO, GK, EK, WE, SK, CN, LO, RM, and SG have contributed equally to this work. LA shares last and senior authorship.
